# The predictive value *of TyG* index for ischemic stroke in patients undergoing maintenance hemodialysis

**DOI:** 10.3389/fmed.2025.1584674

**Published:** 2025-05-30

**Authors:** Yaqing Wang, Xiaojie He, Yuqing Wang, Xiaodong Li

**Affiliations:** ^1^Graduate School of Chengde Medical University, Chengde, Hebei, China; ^2^Graduate School of Hebei Medical University, Shijiazhuang, Hebei, China; ^3^Department of Nephrology, Baoding No. 1 Central Hospital of Hebei Medical University, Baoding, Hebei, China

**Keywords:** Chronic Kidney Disease, hemodialysis, ischemic stroke, insulin resistance, TyG index

## Abstract

**Objectives:**

Triglyceride-glucose (TyG) index is strongly correlated with insulin resistance (IR). A plethora of studies has established the role of TyG index in cerebrovascular diseases. However, the predictive value of TyG index for new-onset ischemic stroke (IS) in maintenance hemodialysis (MHD) patients remains unclear. This study aims to explore the correlation between TyG index and the occurrence of IS in MHD patients.

**Methods:**

This study analyzed clinical data and cranial Computed Tomography results of patients undergoing MHD at Baoding First Central Hospital from January 2019 to January 2024. TyG index was calculated using fasting blood glucose and triglyceride levels. Univariate Logistic regression analysis was employed to identify factors associated with IS, incorporating variables with *P* < 0.05 into multivariate Logistic regression analysis, with results expressed as odds ratio (OR) and 95% confidence interval (95% CI). Receiver operating characteristic (ROC) curve analysis was used to evaluate the predictive value of TyG index for the occurrence of IS.

**Results:**

Triglyceride-glucose index in the IS group was significantly higher than that in the non-IS group, with a statistically significant difference [9.32 (8.87, 9.92) vs 8.59 (8.06, 9.12), *P* < 0.001]. Variables with *P* < 0.05 from the univariate Logistic regression analysis, along with clinically relevant variables, were included in the multivariate Logistic regression analysis, indicating that an elevated TyG index is a factor associated with IS in hemodialysis patients (OR = 2.09, 95% CI 1.073–3.781, *P* < 0.001). ROC curve analysis revealed that the optimal cutoff value of TyG index for diagnosing new-onset IS in MHD patients was 9.2, with an area under the curve of 0.75 (95% CI 0.70–0.79, *P* < 0.001). Restricted cubic spline analysis demonstrated a non-linear relationship between TyG index and risk (non-linear *p* = 0.010).

**Conclusion:**

Triglyceride-glucose index is significantly elevated in MHD patients with new-onset IS, serving as a potential risk factor for such events and offering valuable clinical diagnostic reference.

## 1 Introduction

Chronic Kidney Disease (CKD) is a progressive, irreversible condition that presents a substantial public health challenge. The global prevalence of CKD is estimated to be between 10% and 13% (1). CKD is defined by the presence of kidney damage or a sustained estimated glomerular filtration rate of less than 60 ml/min/1.73 m^2^ for 3 months or longer. Kidney damage is typically ascertained through albuminuria but may also encompass abnormalities in urine sediment, pathology, or imaging studies, as well as acid-base and electrolyte disturbances due to tubular dysfunction or a history of kidney transplantation (2, 3). When CKD progresses to End Stage Renal Disease, renal function is insufficient to sustain life, necessitating Renal Replacement Therapy (RRT) (4). Hemodialysis (HD) is the most prevalent form of RRT worldwide (5).

Patients undergoing Maintenance Hemodialysis (MHD) are at an elevated risk of cardio cerebrovascular events, with ischemic stroke (IS) being one of the most common complications (6). Despite advancements in HD technology, MHD patients face 7.1-fold higher risk of IS compared to healthy individuals (7). The mechanisms underlying IS in MHD patients are exceedingly complex, involving a range of dialysis-related factors such as hemodynamic alterations, the impact of arteriovenous fistulas, dialysis-related amyloidosis, vascular calcification, and the effects of dialysis fluid (8).

Furthermore, insulin resistance (IR) plays a pivotal role in the development of IS. The hyperinsulinemic-euglycemic clamp technique is regarded as the definitive method for evaluating IR. Nevertheless, its complexity, high expenses, and ethical concerns limit its broad clinical application. In recent years, researchers have proposed Triglyceride Glucose Index (TyG) as an important indicator of IR, demonstrating a strong correlation with the Homeostasis Model Assessment of Insulin Resistance and the hyperinsulinemic-euglycemic clamp technique (9, 10). Numerous studies have established that TyG index can serve as a novel marker of IR and is associated with atherosclerosis and the occurrence of acute lacunar stroke (11, 12). However, no existing research has explored the relationship between TyG index and the risk of IS in MHD patients.

Therefore, this study aims to retrospectively examine whether TyG index is a factor influencing new-onset IS in MHD patients, with the goal of mitigating stroke incidence in this population and providing a theoretical basis for individualized stratified management of MHD patients.

## 2 Materials and methods

This study focuses on patients undergoing HD at Baoding No. 1 Central Hospital between January 2019 and January 2024. Patients were categorized into an IS group or a non-IS group based on whether they experienced their first incidence of IS during dialysis. All IS diagnoses were substantiated through clinical symptoms and cranial Computed Tomography (CT) scans, in accordance with the diagnostic criteria for cerebral infarction outlined in the “Guidelines for the Early Management of Patients With Acute Ischemic Stroke: 2019 Update to the 2018 Guidelines for the Early Management of Acute Ischemic Stroke” (13). The diagnoses were made by two neurologists based on clinical data, with a third neurologist assessing cases where there was disagreement.

### 2.1 Inclusion criteria

① Individuals aged 18 years and older, regardless of gender;

② Patients receiving MHD for at least 3 months;

③ No known history of IS at the time of enrollment;

④ At least one cranial CT scan performed post-onset for definitive diagnosis;

### 2.2 Exclusion criteria

① Patients with severe primary diseases of vital organs such as the heart and lungs, or those with organ failure and severe functional impairments;

② Patients with a history of previous IS;

③ Patients with active or metastatic tumors;

④ Patients who have undergone major surgery, suffered severe trauma, or have severe infections;

⑤ Incomplete clinical data;

⑥ Pregnant or lactating women.

**FIGURE 1:**
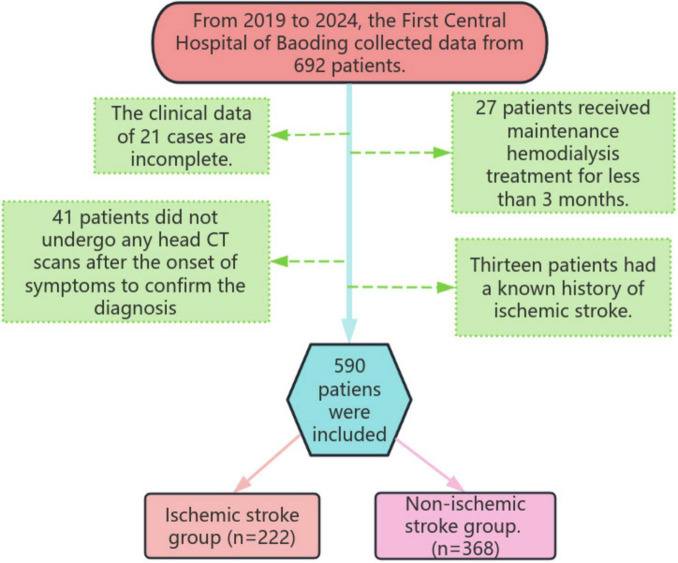
Research flowchart.

Ultimately, a total of 590 patients were included in this study, and they were divided into the IS group and the non-IS group based on the occurrence of new-onset IS during the dialysis period (Figure 1).

### 2.3 Data collection

① Demographic indicators: age, gender, body mass index (BMI), smoking history, alcohol consumption history;

② Clinical and laboratory indicators: hypertension (HTN), diabetes, hemoglobin, platelets, D-dimer, glucose level (fasting plasma glucose), albumin, fibrinogen, creatinine, uric acid, triglycerides, total cholesterol, low-density lipoprotein high-density lipoprotein, serum calcium, serum phosphorus. Demographic information, clinical characteristics, and laboratory test results at admission were obtained from electronic medical records. Cranial CT scans were performed by specialized radiologists, and imaging reports were written by imaging physicians specializing in CT.

### 2.4 TyG assessment

The TyG index was calculated using the following formula: TyG = ln[TG (mg/dL)*FBG (mg/dL)]/2.

### 2.5 Statistical analysis

A comprehensive database was created using Microsoft Excel software for medical records. Data analysis was conducted using SPSS 26.0 and R programming for statistical evaluation. Normally distributed continuous variables were expressed as mean ± standard deviation, and independent sample *t*-tests were used for comparisons between groups. Non-normally distributed continuous variables were expressed as median (*P*25, *P*75), and rank-sum tests were used for comparisons between groups. Categorical variables were expressed as frequency (%), and chi-square (χ^2^) tests were used for comparisons between groups. Initially, differences in categorical and continuous variable characteristics were compared between the IS group and the non-IS group. Variables with *P* < 0.05 were included in multivariate logistic regression to control for confounding factors and further screen for independent variables associated with new-onset IS. TyG index was divided into quartiles, and logistic regression analysis was used to further evaluate the correlation between TyG index and the risk of new-onset IS in MHD patients. The results of various models, adjusted for confounders, were presented as odds ratios (OR) with 95% confidence intervals (CI). Model 1 included only TyG, Model 2 adjusted for age and gender, and Model 3 further adjusted for various potential confounding factors related to clinical outcomes of IS. The relationship between TyG and IS was evaluated using restricted cubic splines (RCS). Stratified logistic regression models were applied to investigate interactions between several subgroups at the stratified level. Receiver operating characteristic (ROC) curve analysis was used to plot the effectiveness of triglycerides, fasting plasma glucose, and TyG in relation to IS, and the area under the ROC curve was calculated to assess predictive value. *P* < 0.05 was considered statistically significant.

### 2.6 Ethics statement

This study was approved by the Ethics Committee of Baoding No. 1 Central Hospital (Approval Number: 2024244) and conducted in accordance with the relevant guidelines and regulations of the Helsinki Declaration.

## 3 Results

### 3.1 Baseline characteristics

A comparative analysis of baseline characteristics and test results between IS and non-IS groups in MHD patients was conducted. A total of 692 patients were initially screened, with 102 cases excluded, leaving 590 patients in this single-center study. Patients were categorized into the IS group (*n* = 222) and the non-IS group (*n* = 368) based on the occurrence of IS during dialysis treatment. Regarding baseline characteristics, a higher percentage of patients in the IS group had HTN compared to the non-stroke group. Additionally, levels of triglycerides, low-density lipoprotein, and TyG index were significantly elevated in the IS group. Notably, the IS group exhibited younger age and lower fibrinogen levels compared to the non-IS group, which could be influenced by selection bias due to the study’s single-center design and limited sample size. These differences were statistically significant (*P* < 0.05) (Table 1).

**TABLE 1:** Demographic data and baseline characteristics of two groups.

Variables	Total (*n* = 590)	Non-IS group (*n* = 368)	IS group (*n* = 222)	Statistic	*P*
Gender				χ^2^ = 0.10	0.748
Female	308 (52.20)	194 (52.72)	114 (51.35)	–	–
Male	282 (47.80)	174 (47.28)	108 (48.65)	–	–
Smoke				χ^2^ = 3.42	0.064
No	240 (40.68)	139 (37.77)	101 (45.50)	–	–
Yes	350 (59.32)	229 (62.23)	121 (54.50)	–	–
Drink				χ^2^ = 1.97	0.160
No	301 (51.02)	196 (53.26)	105 (47.30)	–	–
Yes	289 (48.98)	172 (46.74)	117 (52.70)	–	–
HTN				χ^2^ = 16.00	**< 0.001**
No	66 (11.19)	56 (15.22)	10 (4.50)	–	–
Yes	524 (88.81)	312 (84.78)	212 (95.50)	–	–
DM				χ^2^ = 1.02	0.313
No	324 (54.92)	208 (56.52)	116 (52.25)	–	–
Yes	266 (45.08)	160 (43.48)	106 (47.75)	–	–
Age (years)	70.00 (63.00, 77.00)	71.00 (64.00, 77.00)	68.00 (61.00, 76.00)	Z = –2.67	**0.008**
BMI (kg/m^2^)	23.50 (20.80, 26.10)	23.50 (20.80, 26.30)	23.55 (20.83, 25.58)	Z = –0.75	0.454
HB (g/L)	84.00 (69.00, 102.00)	84.00 (68.00, 103.00)	83.50 (69.00, 100.00)	Z = –0.14	0.885
PLT (10^9^/L)	193.00 (146.25, 246.00)	189.00 (146.50, 239.00)	202.50 (146.25, 258.00)	Z = –1.38	0.168
DD (mg/dL)	1.25 (0.68, 2.95)	1.35 (0.59, 3.44)	1.20 (0.75, 1.91)	Z = –0.80	0.426
Fib (g/L)	4.44 (3.45, 5.35)	4.58 (3.59, 5.41)	4.11 (3.36, 5.27)	Z = –2.16	**0.031**
ALB (g/L)	34.60 (30.80, 39.30)	35.00 (31.00, 39.42)	34.20 (30.20, 38.53)	Z = –1.33	0.182
Cr (umol/L)	601.81 (460.66, 783.08)	605.90 (459.51, 790.92)	600.14 (462.80, 762.01)	Z = –0.71	0.475
UA (umol/L)	345.70 (252.38, 427.77)	346.10 (245.92, 427.72)	341.28 (256.67, 429.70)	Z = –0.77	0.442
Ca (mmol/L)	2.19 (1.67, 2.86)	2.29 (1.58, 2.93)	2.08 (1.78, 2.59)	Z = –1.59	0.111
Mg (mmol/L)	1.06 (0.84, 1.27)	1.07 (0.81, 1.29)	1.06 (0.87, 1.24)	Z = –0.14	0.891
Glu (mmol/L)	9.69 (7.14, 12.17)	9.67 (7.47, 11.85)	9.80 (6.80, 12.81)	Z = –0.04	0.968
TG (mmol/L)	1.35 (1.01, 1.68)	1.27 (0.94, 1.55)	1.56 (1.12, 1.89)	Z = –7.10	**< 0.001**
TC (mmol/L)	5.30 (3.30, 7.47)	5.30 (3.30, 7.50)	5.20 (3.30, 7.40)	Z = –0.03	0.978
LDL-c (mmol/L)	2.80 (2.24, 3.31)	2.79 (2.21, 3.24)	2.90 (2.31, 3.52)	Z = –2.34	**0.019**
HDL-c (mmol/L)	3.10 (2.00, 4.10)	3.10 (1.90, 4.10)	3.10 (2.12, 4.30)	Z = –1.15	0.248
TyG	8.88 (8.33, 9.44)	8.59 (8.06, 9.12)	9.32 (8.87, 9.92)	Z = –9.92	**< 0.001**

Bold values indicated statistical significance. HTN, hypertension; DM, diabetes; BMI, body mass index; HB, hemoglobin; PLT, platelets; DD, D-dimer; ALB, albumin; Fib, fibrinoge; Cr, creatinine; UA, uric acid; Ca, calcium; Mg, magnesium; Glu, triglycerides; TG, triglycerides; TC, total cholesterol; LDL-C, low-density lipoprotein cholesterol; HDL-C, high-density lipoprotein cholesterol; TyG, triglyceride-glucose.

#### 3.2 Results of multivariable logistic regression analysis

In a multivariate Logistic regression analysis with the occurrence of IS in HD patients as the dependent variable, statistically significant factors between the two groups were included as independent variables. The results indicated that HTN (*P* = 0.002, OR = 3.20, 95% CI: 1.53–6.68), triglycerides (*P* < 0.001, OR = 3.17, 95% CI: 2.05–4.92), and TyG index (*P* < 0.001, OR = 2.09, 95% CI: 1.67–2.61) were identified as risk factors for the first occurrence of IS in MHD patients (*P* < 0.05) (Table 2).

**TABLE 2:** Results of multivariable logistic regression analysis.

Variables	β	S.E	Z	*P*	OR (95% CI)
HTN					
No	–	–	–	–	1.00 (reference)
Yes	1.16	0.38	3.10	**0.002**	3.20 (1.53∼6.68)
Age	–0.02	0.01	–1.80	0.072	0.98 (0.96∼1.00)
Fib	–0.09	0.06	–1.53	0.127	0.91 (0.81∼1.03)
TG	1.16	0.22	5.17	**< 0.001**	3.17 (2.05∼4.92)
TyG	0.74	0.11	6.45	**< 0.001**	2.09 (1.67∼2.61)

Bold values indicated statistical significance. HTN, hypertension; Fib, fibrinoge; TG, triglycerides; TyG, triglyceride-glucose.

### 3.3 ROC analysis of TyG index for predicting new-onset IS in MHD patients

**FIGURE 2:**
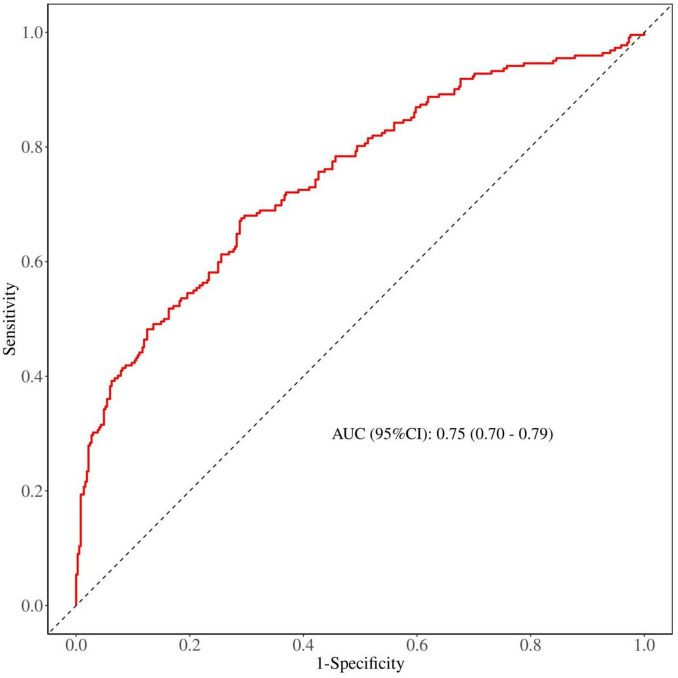
Triglyceride-glucose (TyG) refers to a receiver operating characteristic (ROC) analysis used to predict new ischemic stroke (IS) in patients undergoing maintenance hemodialysis (MHD).

We performed an analysis of the ROC curves for both the IS group and the non-IS group to determine the optimal threshold. Our findings reveal that the area under the curve (AUC) for TyG index in predicting new-onset IS in MHD patients is 0.75 (95% CI: 0.70–0.79, *P* < 0.01); the optimal cutoff value is 9.2; with a sensitivity of 0.80 and specificity of 0.59 (Figure 2 and Table 3).

**TABLE 3:** Predictive value of TyG for IS risk.

AUC (95% CI)	Accuracy (95% CI)	Sensitivity (95% CI)	Specificity (95% CI)	Cut off values
0.75 (0.70–0.79)	0.73 (0.69–0.76)	0.80 (0.76–0.84)	0.59 (0.53–0.66)	9.206

TyG, triglyceride-glucose; IS, ischemic stroke; AUC, area under curve.

### 3.4 Association between TyG and the risk of IS in various models

Multivariate logistic regression analysis was conducted with new-onset IS as the dependent variable and TyG index as the independent variable. In Model 1, without adjusting for any covariates, the third and fourth quartiles of TyG index were identified as risk factors for new-onset IS in patients with MHD compared to the first and second quartiles (*p* < 0.05). In Model 2, after adjusting for gender and age, the results showed that the third and fourth quartiles of TyG index remained risk factors for new-onset IS in MHD patients compared to the first and second quartiles (*p* < 0.05). In Model 3, after further adjusting for HTN and family history of diabetes, the results still indicated that the third and fourth quartiles of TyG index were risk factors for new-onset IS in MHD patients compared to the first and second quartiles. In Model 4, after adjusting for gender, age, HTN, history of diabetes, fasting venous blood glucose, and triglycerides, the results consistently demonstrated that the third and fourth quartiles of TyG index were risk factors for new-onset IS in MHD patients compared to the first and second quartiles (*p* < 0.05) (Table 4).

**TABLE 4:** Patients undergoing MHD with TyG index values in the higher quartiles are at risk of new IS, as indicated by the ORs and 95% CIs.

Variables	Model 1		Model 2		Model 3		Model 4	
	**OR (95% CI)**	***P***	**OR (95% CI)**	***P***	**OR (95% CI)**	***P***	**OR (95% CI)**	***P***
1	1.00 (reference)	–	1.00 (reference)	–	1.00 (reference)	–	1.00 (reference)	–
2	1.35 (0.75∼2.41)	0.316	1.38 (0.77∼2.47)	0.281	1.33 (0.74∼2.39)	0.341	1.13 (0.62∼2.06)	0.685
3	3.81 (2.22∼6.54)	**< 0.001**	3.85 (2.24∼6.64)	**< 0.001**	3.68 (2.13∼6.35)	**< 0.001**	3.13 (1.79∼5.47)	**< 0.001**
4	10.27 (5.92∼17.81)	**< 0.001**	10.27 (5.90∼17.88)	**< 0.001**	10.08 (5.77∼17.59)	**< 0.001**	7.58 (4.18 ∼13.73)	**< 0.001**

Bold values indicated statistical significance. Model 1: unadjusted model. Model 2: adjusted for age, sex. Model 3: adjusted for above + history of HTN + history of diabetes. Model 4: adjusted for above + Glu + TG. MHD, maintenance hemodialysis; TyG, triglyceride-glucose; IS, ischemic stroke; Glu, triglycerides; TG, triglycerides;

### 3.5 Exploration of subgroup analysis

**FIGURE 3:**
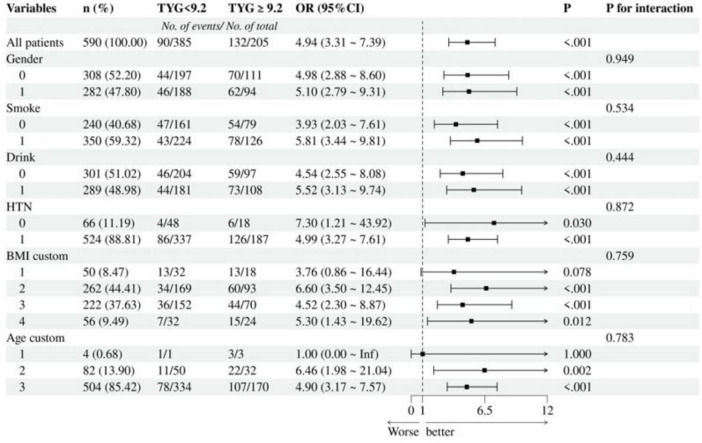
Subgroup analysis of association between TyG and IS risk. TyG, triglyceride-glucose; IS, ischemic stroke; HTN, hypertension; BMI, body mass index.

To further explore interactions among variables, we stratified the samples into a high TyG index group (≥ 9.2) and a low TyG index group (< 9.2) based on the optimal cut-off value, followed by subgroup analysis (Figure 3). The results indicated no significant interactive effects of various factors on the risk of IS. This suggests that TyG itself serves as a factor that increases the risk of IS.

### 3.6 RCSs for analyzing the relationship between TyG and the risk of IS

**FIGURE 4:**
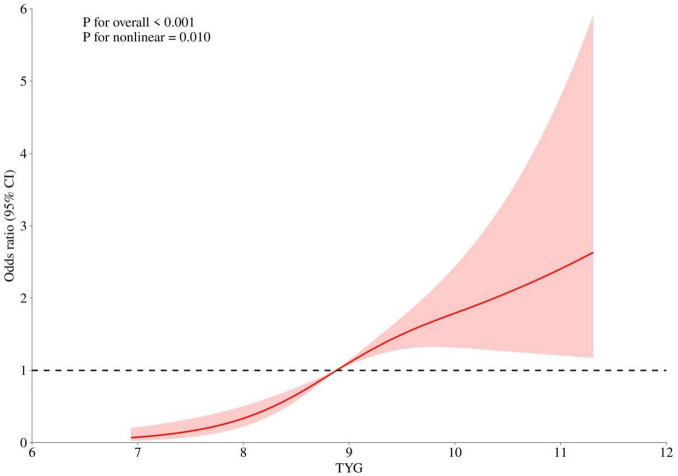
Restricted cubic spline analysis of the relationship between triglyceride-glucose (TyG) and ischemic stroke (IS) risk.

To further validate the association between TyG index and new-onset IS in MHD patients, we employed RCS analysis. Our findings demonstrated a non-linear relationship between TyG index and new-onset IS in MHD patients (Figure 4).

## 4 Discussion

We analyzed data from 590 patients undergoing MHD at our center and identified a positive correlation between baseline TyG index levels and the incidence of new-onset IS in MHD patients. Univariate analysis revealed that hypertensive patients, along with elevated triglycerides, low-density lipoprotein, and TyG index, were more prevalent in the cerebral infarction group compared to the non-cerebral infarction group. Notably, our findings indicated that the mean age and fibrinogen levels were lower in the cerebral infarction group compared to the non-cerebral infarction group. This discrepancy may be due to the single-center design and the relatively small sample size, introducing potential selection bias. Multivariate Logistic regression analysis demonstrated that a history of HTN, elevated triglyceride levels, and a high TyG index were risk factors for cerebral infarction among MHD patients. Furthermore, there was a non-linear relationship between TyG index and the occurrence of IS in MHD patients, with a TyG index greater than 9.2 being associated with a significantly increased risk of IS. This may be because when the level of TyG increases to a certain extent, certain pathological processes within the body may accelerate, such as atherosclerosis and vascular inflammation. The acceleration of these pathological processes may further increase the risk of IS, resulting in an accelerated trend in the association between TyG levels and the incidence of new IS in MHD patients.

Research indicates that dialysis patients of any age are at a high risk of IS, with generally poor prognosis (8, 14). A single-center study conducted in the United Kingdom surveyed 2,384 HD patients and found that the incidence of stroke was 15 cases per 1,000 patient-years. The primary risk factors include diabetes and HTN (15). The primary risk factors encompass diabetes and HTN. A nationwide longitudinal study conducted across dialysis facilities in Japan enrolled 151,350 hemodialysis patients. In this prospective cohort study, 4,967 patients (3.2%) experienced IS within 1 year. This proportion is almost 10 times higher than that observed in the general population (16). The mechanisms underlying IS in MHD patients are highly complex and encompass: (1) several traditional risk factors such as advanced age, diabetes, HTN, hyperlipidemia, obesity, and smoking; (2) uremia-related mechanisms, including chronic inflammation, oxidative stress, anemia, malnutrition, abnormal calcium and phosphorus metabolism, water and sodium retention, and the toxic effects of certain substances; and (3) dialysis-related mechanisms, such as hemodynamic alterations, the impact of arteriovenous fistulas, dialysis-related amyloidosis, vascular calcification, and the influence of dialysis fluid composition (17).

Our research corroborate previous research, identifying HTN as a risk factor for IS. Analysis reveals that HTN may contribute to IS through the following mechanisms: it accelerates atherosclerosis and small artery pathology, leading to smooth muscle cell proliferation and collagen fiber hyalinosis in the media of blood vessel walls, resulting in wall thickening, lumen stenosis or occlusion, and ultimately causing cerebral ischemia or sclerosis. A study in Japan has shown that HTN increases the lifetime risk of IS (18). Large-scale cohort studies have also shown that effective blood pressure control can reduce the incidence of IS (19–22). The majority of patients undergoing HD exhibit poor blood pressure control. This may be due to decreased sodium excretion in renal failure, leading to water and sodium retention, increased blood volume, and subsequently elevated blood pressure. Additionally, uremia directly enhances sympathetic nerve excitability, further contributing to HTN (23). Our study further found that triglyceride levels were higher in the IS group compared to the non-IS group (*P* < 0.05), suggesting that abnormal elevation of triglycerides is involved in the pathogenesis of cerebral infarction. Triglycerides, a type of blood lipid, can damage the arterial intima and accumulate beneath it. Over time, these deposits can form atherosclerotic plaques, obstruct blood vessels, accelerate atherosclerosis, and promote thrombosis. Therefore, blood lipid levels are closely related to the occurrence of cerebral infarction. A Mendelian randomization study by Sun L et al. showed a positive correlation between elevated triglycerides and the occurrence of IS (24). This supports our findings, which align with previous research.

Currently, literature on the relationship between TyG and IS remains limited. Our study represents the first investigation into the predictive value of TyG index for incident IS in MHD patients. Our research indicates that a high TyG index serves as a risk factor for the development of IS in MHD patients. ROC curve analysis revealed an optimal cutoff value of 9.2 for TyG index in diagnosing incident IS in MHD patients, with an AUC of 0.75 (95% CI: 0.70–0.79, *P* < 0.001), suggesting robust predictive value. Consequently, an elevated TyG index emerges as a novel predictor for incident IS in MHD patients. TyG index serves as an alternative indicator for IR, offering a broader range of predictive options compared to traditional stroke risk factors. Furthermore, TyG index is more readily accessible than other insulin resistance indicators such as HOMA-IR. When compared to complex stroke risk models, TyG index represents a simpler and more practical metric for initial stroke risk assessment. Consequently, TyG index holds significant clinical value in predicting stroke in MHD patients.

The mechanisms underlying the relationship between TyG index and IS remain unclear; however, IR may play a pivotal role in this process. IR refers to the decreased efficiency of insulin in facilitating glucose uptake and utilization, serving as a prominent feature of metabolic syndrome and is recognized as one of the critical risk factors for IS. The potential reasons for this are as follows: Firstly, IR leads to the expression of inflammation-related genes, inhibiting the action of insulin signaling at the endothelial cellular level and reducing the response to oxidative stress, thereby inducing chronic inflammation and endothelial dysfunction. This promotes foam cell formation, initiates atherosclerosis, and contributes to the development of vulnerable plaques (25). Additionally, studies suggest that IR can potentiate the mitogen-activated protein kinase signaling pathway, enhancing cell adhesion and interaction, further facilitating atherosclerosis (26). Secondly, platelets play a crucial role in the coagulation process, and when platelet function is deregulated, coagulation may occur inappropriately. Under normal circumstances, insulin reduces platelet aggregation by facilitating intracellular magnesium transport and enhances platelet sensitivity to factors such as prostacyclin (27). However, in a state of IR, this inhibitory effect diminishes, leading to abnormally active platelet activation processes (such as adhesion, activation, and aggregation), which can trigger microvascular and macrovascular events, resulting in vascular stenosis and predisposing patients to ischemic events (27, 28). Lastly, individuals with a higher TyG index typically exhibit a multitude of cerebrovascular disease risk factors, including advanced age, male gender, higher BMI, smoking, and a history of myocardial infarction, diabetes, HTN, hypercoagulability, and hypercholesterolemia. Furthermore, IR can modify and amplify the effects of these risk factors, increasing the probability of IS occurrence (25).

Triglyceride-glucose index is widely regarded as a reliable and direct surrogate marker for IR in clinical practice. Studies have demonstrated a correlation between TyG index and IS in the general population, with the first indication of a linear relationship reported in the literature (29). Our investigation into the association between TyG index and IS in the MHD population revealed a non-linear relationship. Research has demonstrated that even in the absence of traditional risk factors for cardiovascular and cerebrovascular diseases, an elevated TyG index level is associated with an increased risk of these conditions (30, 31). A retrospective analysis by Wu Y et al. found that among adults aged over 45, a consistently high TyG index was associated with a greater risk of IS. Subgroup analysis revealed similar results even among participants without diabetes or dyslipidemia (32). Wang et al. conducted an 11 years follow-up study on 97,653 individuals without a history of IS and found that elevated baseline and long-term cumulative average TyG index levels could predict the occurrence of IS in the general population (33). A meta-analysis involving over 5 million participants found that the TyG index was associated with a 1.3-fold increased risk of IS (34). Moreover, several studies have shown that TyG index is not only associated with IS but also has significant predictive value for disease severity and prognosis (35–37). Thus, it is clear that IR plays a role in the onset of IS, and TyG index, as a reliable and simple indicator of IR, aids in the early identification of high-risk individuals for IS.

The primary advantage of this study lies in our validation that an elevated TyG index is a significant risk factor for the onset of IS in MHD patients. Nevertheless, our study has several limitations. Firstly, as a single-center retrospective study, the sample size is relatively small, necessitating further multi-center research to thoroughly explore the relationship between TyG index and the occurrence of the first ischemic stroke in patients undergoing MHD, as well as its underlying mechanisms. Secondly, despite the conduct of multivariate adjustments, there may still be some unmeasurable confounding factors, such as the patients’ medication use and inflammatory status. For instance, metformin, a diabetes medication, can enhance insulin sensitivity, while statins may influence triglyceride levels. Changes in the levels of inflammatory markers such as C-reactive protein may alter insulin sensitivity and subsequently impact lipid metabolism. Future studies should incorporate more potential confounding factors to enhance the accuracy of the research. Lastly, our study focused solely on the baseline TyG index, neglecting its dynamic fluctuations throughout hospitalization. Various factors, including diet, exercise, and pre-testing mood, could potentially affect TyG index. Consequently, future research should prioritize monitoring and analyzing the dynamic variations of TyG index to gain a more comprehensive understanding of its clinical utility.

## 5 Conclusion

Our findings suggest that TyG index is an economical and valuable biomarker for predicting new-onset IS in MHD patients. We also discovered a non-linear relationship between TyG index and new IS in MHD patients. Therefore, we recommend monitoring the TyG index in MHD patients to assess their risk of IS.

## Data Availability

The raw data supporting the conclusions of this article will be made available by the authors, without undue reservation.
