# Supplementary figures and images for: The predictive value of TyG index for ischemic stroke in patients undergoing maintenance hemodialysis
Source: Front Med (Lausanne). 2025 May 30;12:1584674. doi: 10.3389/fmed.2025.1584674 (PMC12162943; doi:10.3389/fmed.2025.1584674)

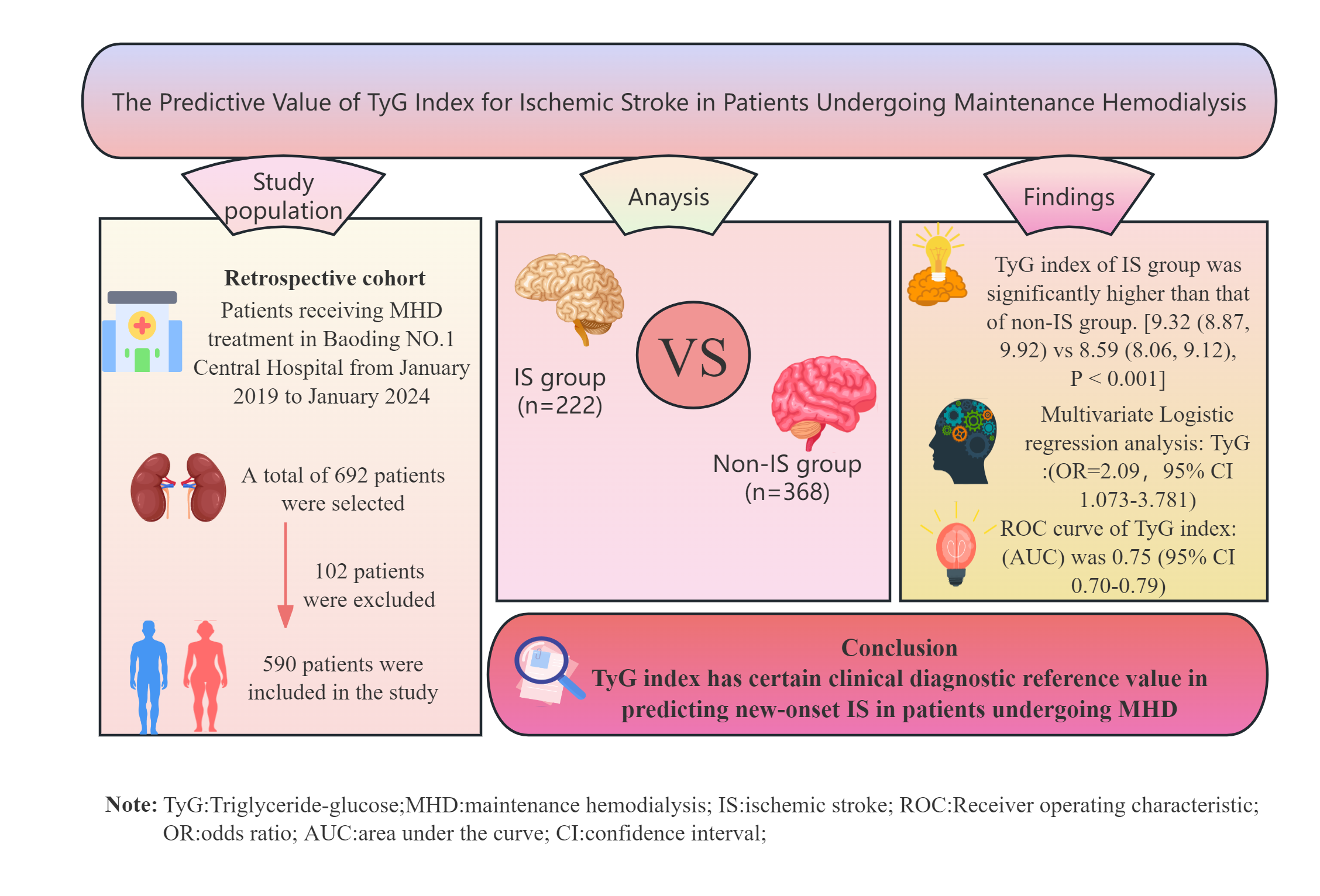

Supplement: Supplementary file 1 [file Image_1.tif]
